# A Case of Corneal Endothelial Cell Loss After PreserFlo MicroShunt Implantation Requiring Device Removal and Ahmed Glaucoma Valve Implantation With Tube Insertion Into the Vitreous Cavity

**DOI:** 10.7759/cureus.85078

**Published:** 2025-05-30

**Authors:** Tomoyuki Watanabe, Koki Honzawa, Hisato Gunji, Hiroshi Horiguchi, Tadashi Nakano

**Affiliations:** 1 Department of Ophthalmology, The Jikei University School of Medicine, Tokyo, JPN

**Keywords:** ahmed glaucoma valve, corneal endothelial cell loss, intraocular pressure, preserflo microshunt, ripasudil

## Abstract

We report a case of progressive endothelial cell loss (ECL) following PreserFlo MicroShunt (PMS) (Santen Pharmaceutical, Osaka, Japan) implantation, which was successfully managed by PMS removal and the pars plana placement of Ahmed glaucoma valve (AGV) implantation. An 82-year-old man with primary open-angle glaucoma underwent PMS implantation combined with cataract surgery. Preoperative endothelial cell density (ECD) was 2,148 cells/mm², and intraocular pressure (IOP) was 21 mmHg despite five topical IOP-lowering medications. One week postoperatively, the IOP normalized to 11 mmHg without medication, but the ECD decreased to 1,400 cells/mm². Over the following 12 months, the ECD progressively declined to 880 cells/mm². PMS removal and AGV implantation, with tube insertion into the vitreous cavity, were performed to prevent further ECL and maintain IOP control. After the second surgery, IOP stabilized between 10 and 14 mmHg with topical treatment, including ripasudil, and ECD remained stable at approximately 1,200 cells/mm². This case demonstrates the potential for significant and progressive ECL following PMS implantation and highlights that timely intervention with PMS removal and AGV implantation with tube insertion into the vitreous cavity can effectively stabilize ECD and achieve adequate IOP control, emphasizing the importance of close postoperative monitoring and the prompt management of complications.

## Introduction

The PreserFlo MicroShunt (PMS) (Santen Pharmaceutical, Osaka, Japan) is a minimally invasive glaucoma drainage device designed to reduce intraocular pressure (IOP) by shunting the aqueous humor from the anterior chamber to the subconjunctival space. PMS is considered relatively safe compared to traditional trabeculectomy because it has been demonstrated to reduce the incidence of hypotony and corneal endothelial cell loss (ECL) [1,2]. However, understanding and managing potential complications are essential. Among these, ECL following PMS implantation has been reported, although documented cases remain limited [3,4]. The precise mechanisms underlying ECL after PMS implantation and appropriate management strategies remain poorly understood. Previous reports have focused on PMS removal alone, raising concerns about maintaining adequate IOP control post surgery.

This case report aims to raise awareness of ECL after PMS implantation and provide insights into effective management approaches, including the use of alternative glaucoma therapies.

## Case presentation

Initial presentation

We present the case of an 82-year-old man with primary open-angle glaucoma and a medical history of benign prostatic hyperplasia. The right eye was selected for surgical intervention.

Preoperatively, the best-corrected visual acuity (BCVA) of the affected eye was 1.0 (decimal). The IOP was 21 mmHg despite the use of five topical medications: ripasudil, a latanoprost-carteolol analog fixed combination, and a brinzolamide-brimonidine fixed combination. The Humphrey visual field 24-2 Swedish interactive thresholding algorithm (SITA) standard revealed a mean deviation of -10.00 dB in the affected eye, with documented progression over the preceding 18 months. The preoperative central corneal endothelial cell density (ECD), measured by specular microscopy, was 2,148 cells/mm². The preoperative cataract classification was determined as Emery-Little classification grade 2, and the anterior chamber depth was measured at 3.5 mm.

The left eye had an IOP of 16 mmHg with topical medications, a BCVA of 1.2 (decimal), an ECD of 1,661 cells/mm², and a mean deviation of -6.21 dB on visual field testing. No signs of corneal pathology or viral infection were observed in either eye.

PreserFlo MicroShunt implantation combined with cataract surgery and follow-up

**Figure 1:**
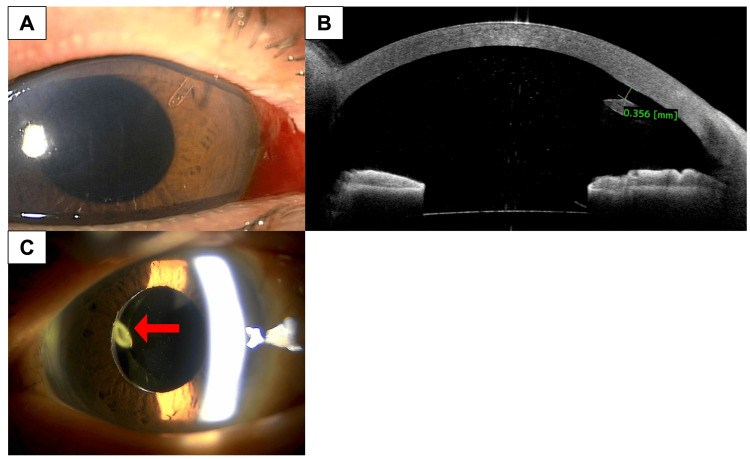
Slit-lamp photograph and anterior segment optical coherence tomography of the right eye after PreserFlo MicroShunt implantation. (A) Slit-lamp photograph showing the PreserFlo MicroShunt (PMS) tube placed in the anterior chamber. (B) Anterior segment optical coherence tomography showing the PMS tube tip positioned 0.36 mm from the corneal endothelium. No hyperreflective lesions suggestive of inflammation were observed. (C) Slit-lamp photograph showing the absence of the PMS and Ahmed glaucoma valve (AGV) in the vitreous cavity. The arrow indicates the tip of the AGV tube.

**Figure 2:**
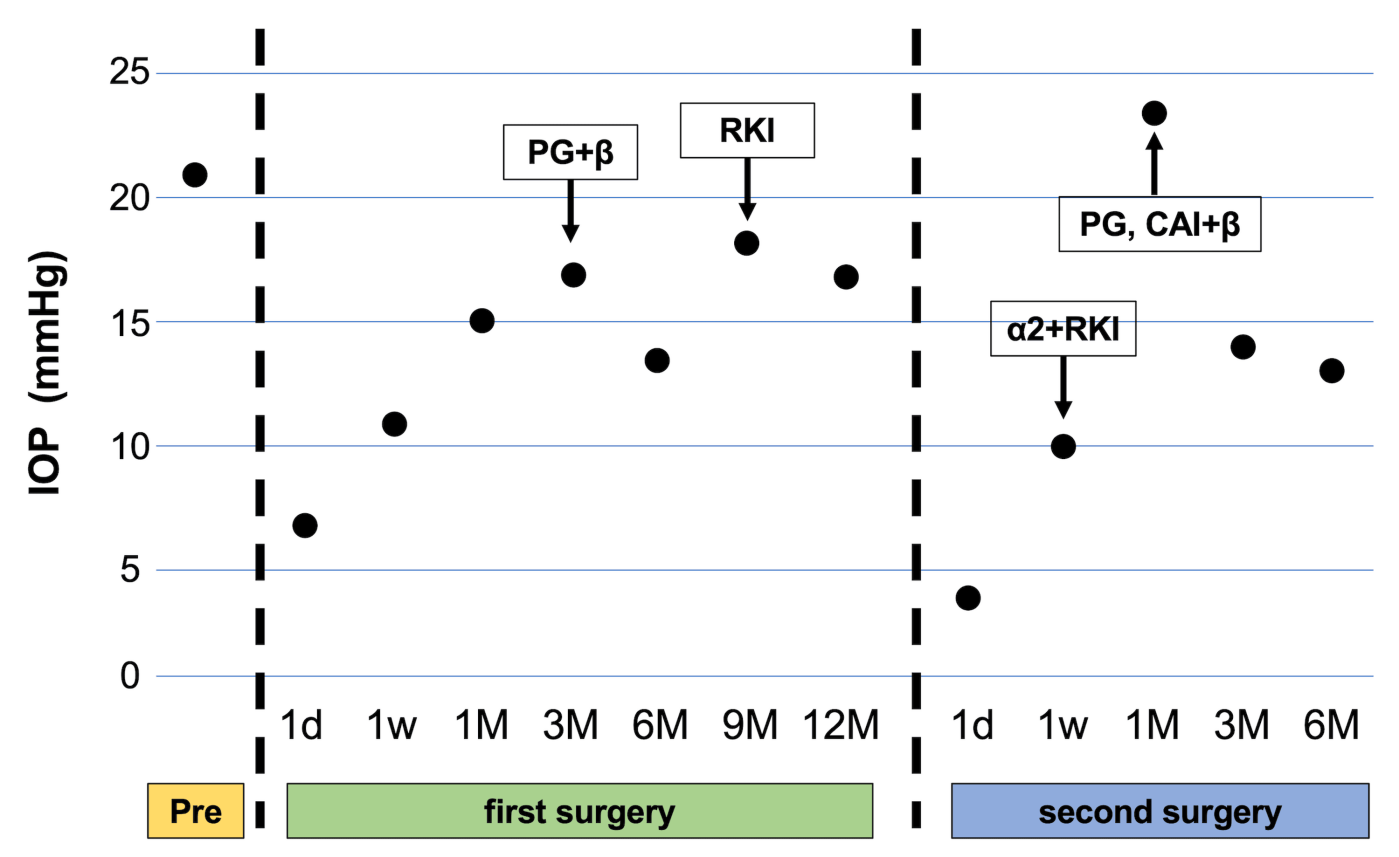
Changes in intraocular pressure before and after surgery. Preoperatively, the intraocular pressure (IOP) was 21 mmHg despite treatment with five different topical medications. On postoperative day 1, the IOP dropped to 7 mmHg but gradually increased to 17 mmHg by postoperative month 3, necessitating the reinitiation of a latanoprost-carteolol analog fixed combination (PG+β). By postoperative month 9, the IOP had risen again to 18 mmHg, prompting the addition of ripasudil (RKI) eye drops. Following the second surgery, the IOP was 4 mmHg on postoperative day 1, increasing to 10 mmHg by postoperative week 1, at which point brimonidine-ripasudil fixed-dose combination (α2+RKI) therapy was started. By postoperative month 1, the IOP had risen to 24 mmHg, requiring the addition of dorzolamide-timolol fixed combination (CAI+β) and latanoprost (PG) eye drops. The IOP subsequently stabilized below 15 mmHg from postoperative month 3 onward.

**Figure 3:**
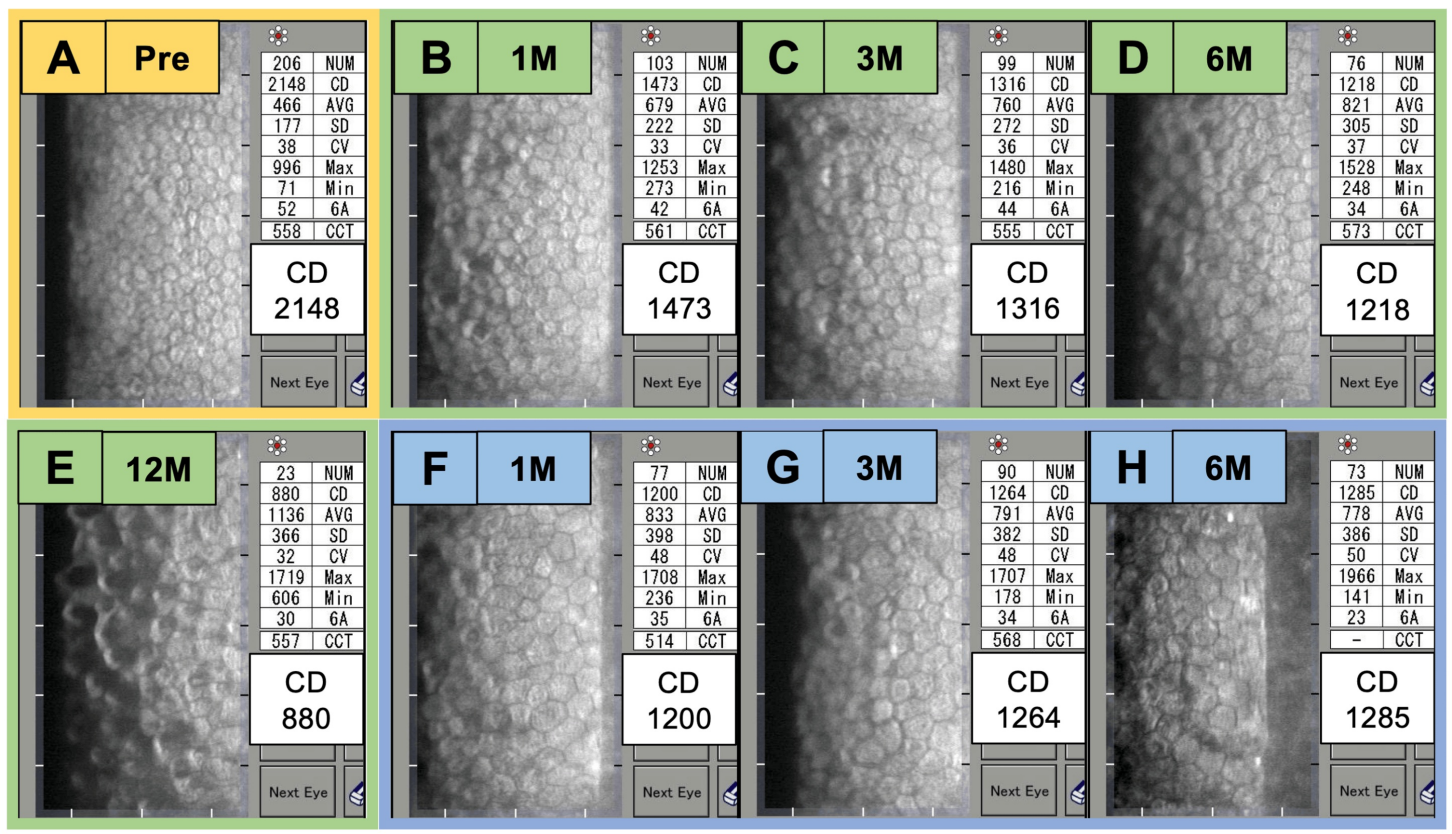
Changes of central corneal endothelial cell density before and after surgery. Specular microscopy showing corneal endothelial cell density (ECD) at various time points: preoperatively (A); at one month (B), three months (C), six months (D), and 12 months (E) after the first surgery; and at one month (F), three months (G), and six months (H) after the second surgery. After the first surgery, ECD decreased to 880 cells/mm² by 12 months. Following the second surgery, the ECD stabilized at approximately 1,200 cells/mm².

The patient underwent cataract surgery and superonasal PMS implantation with mitomycin C (MMC) (0.4 mg/mL) applied for three minutes. After the application, the surgical field was thoroughly irrigated with 150 mL of balanced salt solution to prevent any potential diffusion of mitomycin C into the anterior chamber. We used Healon® (Johnson & Johnson Vision, Jacksonville, FL), which is a cohesive ophthalmic viscosurgical device (OVD), and no other OVDs were used. Cataract surgery was completed within four minutes through a 2.4 mm superotemporal corneal main incision. The PMS tube was positioned in the anterior chamber (Figure 1A), and IOP was normalized to 11 mmHg without medication at postoperative week 1 (Figure 2). However, a marked decrease in central ECD was observed, dropping to 1,400 cells/mm² (Figure 3). Anterior segment optical coherence tomography (AS-OCT) showed no evidence of contact between the device and the corneal endothelium, and no hyperreflective structures were seen around the tube, indicating a lack of inflammation (Figure 1B). The tube's position was assessed, revealing a distance of 0.36 mm between the PMS tube tip and the corneal endothelium, with endothelium edema observed near the PMS tube (Figure 1B). The BCVA remained stable at 1.0. At postoperative month 6, the central ECD further decreased to 1,218 cells/mm², and by postoperative month 12, it had significantly declined to 880 cells/mm² (Figure 3). Despite this ECL, the patient reported no symptoms of pain or vision loss.

PreserFlo MicroShunt removal and Ahmed glaucoma valve (AGV) implantation

Given the significant and progressive ECL, the PMS was removed 12 months after implantation. To maintain IOP control while minimizing further ECL, an Ahmed glaucoma valve (AGV) was implanted superotemporally with the tube inserted into the vitreous cavity during the same surgical session, accompanied by pars plana vitrectomy (Figure 1C). At postoperative week 1, the IOP was well-controlled at 10 mmHg without medication (Figure 2). To prevent an increase in IOP, brimonidine-ripasudil fixed-dose combination eye drops were initiated. However, at postoperative month 1, the IOP increased to 23 mmHg, necessitating the addition of a dorzolamide-timolol fixed combination and latanoprost eye drops. Subsequently, the IOP was successfully reduced to 14 mmHg at postoperative month 3 and remained stable at 13 mmHg at postoperative month 6. Following the second surgery, the central ECD stabilized at approximately 1,200 cells/mm² (Figure 3).

The patient reported no discomfort, and the BCVA remained stable throughout the follow-up period. No postoperative complications, such as inflammation, pain, or device-related issues, were observed throughout the follow-up period.

## Discussion

This case report highlights the complications of ECL following PMS implantation and explores treatment options when ECL develops. The patient experienced rapid and significant ECL following cataract surgery and PMS implantation.

Reports of ECL after PMS implantation are rare. Anton et al. observed no significant ECL over a 20-month follow-up period [5], while Ibarz-Barberá et al. reported approximately 7.4% ECL within one year [4]. The authors compared outcomes between a PMS-only group and a PMS combined with phacoemulsification (PMS+phaco) group. While the PMS+phaco group exhibited higher ECL immediately after surgery, the ECD stabilized afterward, with no significant differences in ECL between the two groups. In the present case, however, the ECL persisted immediately after surgery and throughout the postoperative course, ultimately necessitating device removal.

Previous studies have proposed factors such as tube-endothelium contact and inflammation around the tube as causes of ECL [3]. Additionally, the high fluid flow resulting from the close proximity of the tube tip to the corneal endothelium has been hypothesized to contribute to ECL. Greater ECL was reported when the PMS tube tip was positioned less than 200 μm from the corneal endothelium compared to distances over 500 μm [4]. In this case, the tube-endothelium distance was approximately 360 μm, which exceeds the commonly cited 200 μm threshold considered a potential risk factor for endothelial damage. Nevertheless, ECL may still occur in predisposed patients even at this distance, and this factor likely contributed to the observed ECL. Although no apparent mechanical contact between the tube and endothelium was observed intraoperatively, the possibility of subclinical trauma cannot be entirely excluded. Additionally, intraoperative endothelial injury due to turbulence from fluid dynamics or transient instrument contact during phacoemulsification may have contributed to the early ECD decline.

When ECL occurs, early device removal and the consideration of alternative treatments are essential. In previous studies, PMS removal was performed to prevent further ECL [3,4]. In our case, we also took proactive measures to address the potential for subsequent IOP elevation. Glaucoma surgeries are associated with ECL. Trabeculectomy has been reported to reduce ECD by 6% at 12 months [6] and 9.3% at 24 months [7]. The Baerveldt glaucoma implant results in a 36.8% reduction in ECD over five years [8]. For AGV, ECL severity depends on tube insertion location, with anterior chamber insertion causing greater ECL than ciliary sulcus insertion [9]. Conversely, studies of tube insertion into the vitreous cavity have shown no significant postoperative reduction in ECD [10,11]. Although vitreous cavity tube insertion necessitates vitrectomy and potentially increases the risk of complications, it causes less damage to the corneal endothelium. In this case, PMS removal and AGV implantation were performed on the same day, which successfully normalized the IOP and stabilized the ECD. This represents a unique case of PMS removal and the simultaneous implantation of another glaucoma drainage device in a patient with significant ECL. Our approach may offer an effective treatment option for patients who experience ECL after PMS implantation.

Additionally, the potential role of ripasudil in promoting corneal endothelial recovery should be considered. After the second surgery, a ripasudil-brimonidine fixed-dose combination was used as part of the topical therapy to lower IOP. Beyond its IOP-lowering effects, ripasudil has been shown to facilitate the treatment of corneal endothelial damage by reactivating cell proliferation and migration, as well as restoring endothelial pump and barrier function [12]. In fact, reports have documented the successful treatment of Fuchs' endothelial dystrophy and endothelial damage following cataract surgery using ripasudil eye drops [12,13]. In this case, it is conceivable that ripasudil contributed to stabilizing the corneal ECD.

In this case, PMS removal was performed after significant postoperative progression of the ECL. However, the optimal timing for PMS removal after ECL onset remains unclear. Large-scale studies are needed to elucidate the optimal timing for PMS removal in such cases.

## Conclusions

This case highlights a rare instance of significantly progressive ECL following PMS implantation. Progressive ECL may occur even when the PMS tube is positioned at a seemingly safe distance from the corneal endothelium. Moreover, endothelial cell loss may result not only from PMS placement but also from intraoperative trauma, the additive effect of phacoemulsification, or the potential toxicity of adjunctive agents such as MMC used during surgery. These findings underscore the importance of timely PMS removal and AGV implantation as potential strategies for mitigating endothelial damage and ensuring sustained IOP control in patients with progressive ECL.
